# Administration of Tramadol or Buprenorphine via the drinking water for post-operative analgesia in a mouse-osteotomy model

**DOI:** 10.1038/s41598-019-47186-5

**Published:** 2019-07-24

**Authors:** Paulin Jirkof, Mattea Durst, Robert Klopfleisch, Rupert Palme, Christa Thöne-Reineke, Frank Buttgereit, Katharina Schmidt-Bleek, Annemarie Lang

**Affiliations:** 1 Division of Surgical Research, University Hospital Zurich, University of Zurich, Zurich, Switzerland; 2 Charité – Universitätsmedizin Berlin, corporate member of Freie Universität Berlin, Humboldt-Universität zu Berlin, and Berlin Institute of Health, Department of Rheumatology and Clinical Immunology, Berlin, Germany; 3 Institute of Veterinary Pathology, Department of Veterinary Medicine, Freie Universität Berlin, Berlin, Germany; 4 Unit of Physiology, Pathophysiology and Experimental Endocrinology, Department of Biomedical Sciences, University of Veterinary Medicine, Vienna, Austria; 5 Institute of Animal Welfare, Animal Behavior and Laboratory Animal Science, Department of Veterinary Medicine, Freie Universität Berlin, Berlin, Germany; 6 German Rheumatism Research Centre (DRFZ) Berlin, a Leibniz Institute, Berlin, Germany; 7 Charité – Universitätsmedizin Berlin, corporate member of Freie Universität Berlin, Humboldt-Universität zu Berlin, and Berlin Institute of Health, Julius Wolff Institute and Center for Musculoskeletal Surgery, Berlin, Germany; 8 Charité – Universitätsmedizin Berlin, corporate member of Freie Universität Berlin, Humboldt-Universität zu Berlin, and Berlin Institute of Health Berlin Brandenburg Center for Regenerative Therapies, Berlin, Germany

**Keywords:** Bone, Experimental models of disease, Behavioural methods, Animal behaviour, Animal disease models

## Abstract

Adequate analgesia is essential whenever pain might occur in animal experiments. Unfortunately, the selection of suitable analgesics for mice in bone-linked models is limited. Here, we evaluated two analgesics – Tramadol [0.1 mg/ml (T_low_) vs. 1 mg/ml (T_high_)] and Buprenorphine (Bup; 0.009 mg/ml) – after a pre-surgical injection of Buprenorphine, in a mouse-osteotomy model. The aim of this study was to verify the efficacy of these opioids in alleviating pain-related behaviors, to provide evidence for adequate dosages and to examine potential side effects. High concentrations of Tramadol affected water intake, drinking frequency, food intake and body weight negatively in the first 2–3 days post-osteotomy, while home cage activity was comparable between all groups. General wellbeing parameters were strongly influenced by anesthesia and analgesics. Model-specific pain parameters did not indicate more effective pain relief at high concentrations of Tramadol. In addition, *ex vivo* high-resolution micro computed tomography (µCT) analysis and histology analyzing bone healing outcomes showed no differences between analgesic groups with respect to newly formed mineralized bone, cartilage and vessels. Our results show that high concentrations of Tramadol do not improve pain relief compared to low dosage Tramadol and Buprenorphine, but rather negatively affect animal wellbeing.

## Introduction

Untreated or insufficiently treated pain hampers animal welfare and may have diverse and uncontrollable effects on an organism, such as impaired wound healing, blood flow disorders, or immunosuppression^1–3^. Therefore, adequate pain management is essential for ethical and scientific reasons whenever pain might occur in animal experiments. Unfortunately, data on the efficacy of specific analgesic treatments in commonly used surgical mouse models is still scarce^2,4^.

Human patients with fractures are exposed to experiences such as injury and pain^5^. Acute pain  ranging from moderate to severe is observed in emergency departments when patients arrive with e.g. long bone fractures^6^. In rodent models of closed femur fractures with a stable pin fixation, the pain peak is expected at day 2 post-operatively and might be comparable to the pain experienced by human patients^7^. Even though the acute pain in a fracture is reduced by manual fixation in the animal model, the impact catalogue of the EU-Directive 2010/63/EU classifies stable osteotomies as moderately painful procedures; therefore, reliable analgesic treatment is essential in these models.

Unfortunately, the selection of suitable analgesics for mice in bone healing research is limited, since concerns have been raised about potential adverse effects of non-steroidal anti-inflammatory drugs (NSAIDs) on the initial, inflammatory phase of bone healing^8,9^.

The opioid Buprenorphine and the opioid analogue Tramadol are applied widely in many fields of research, although empirical data on effective doses for rats and mice are rare, and thus dosage recommendations vary substantially in the literature. Buprenorphine is a commonly used fast acting and potent opioid that is mainly administered s.c. or i.p. in dosages of 0.05–0.75 mg/kg every 12 h although recent studies indicate the short half-life and recommend the administration at every 4–6 h^10^. Tramadol, for example, is used in high dosages for bone cancer models (>50–100 mg/kg s.c.)^11^, but appears also effective in lower concentrations to treat acute pain^12^. The application of Tramadol via injections is questionable due to its short half-life of 1–2 h^13,14^, which should be taken into account when designing studies on the efficacy of Tramadol in order to avoid drawing misleading conclusions^15^. Concentrations recommended for oral administration of Tramadol range from 0.025 mg/ml up to 1 mg/ml^16–20^. Nevertheless, to our knowledge, no evidence-based recommendations exist for the treatment of osteotomy pain with Tramadol or Buprenorphine. Therefore, the need for evidence-based and empirical data on the dosage and effectiveness of analgesics in bone research is apparent and further studies must be enrolled.

Here, we performed a refinement study embedded in a basic research study of a mouse-osteotomy model^21^. We evaluated two commonly used pain management protocols, Tramadol (0.1 mg/ml = T_low_ vs. 1 mg/ml = T_high_) and Buprenorphine (0.009 mg/ml = Bup) administered via drinking water, after an initial pre-operative injection of Buprenorphine (0.03 mg/kg, s.c.), for their efficacy and side effects on experimental readouts in a mouse-osteotomy model. We asked the research question whether the application of Tramadol or Buprenorphine in commonly used dosages via drinking water represents a continuous, stress-free method of administering effective analgesia in the mouse-osteotomy model with no adverse impact on fracture healing in the model used.

## Results

### Drinking frequency and water intake are affected by type and concentration of opioid

**Figure 1:**
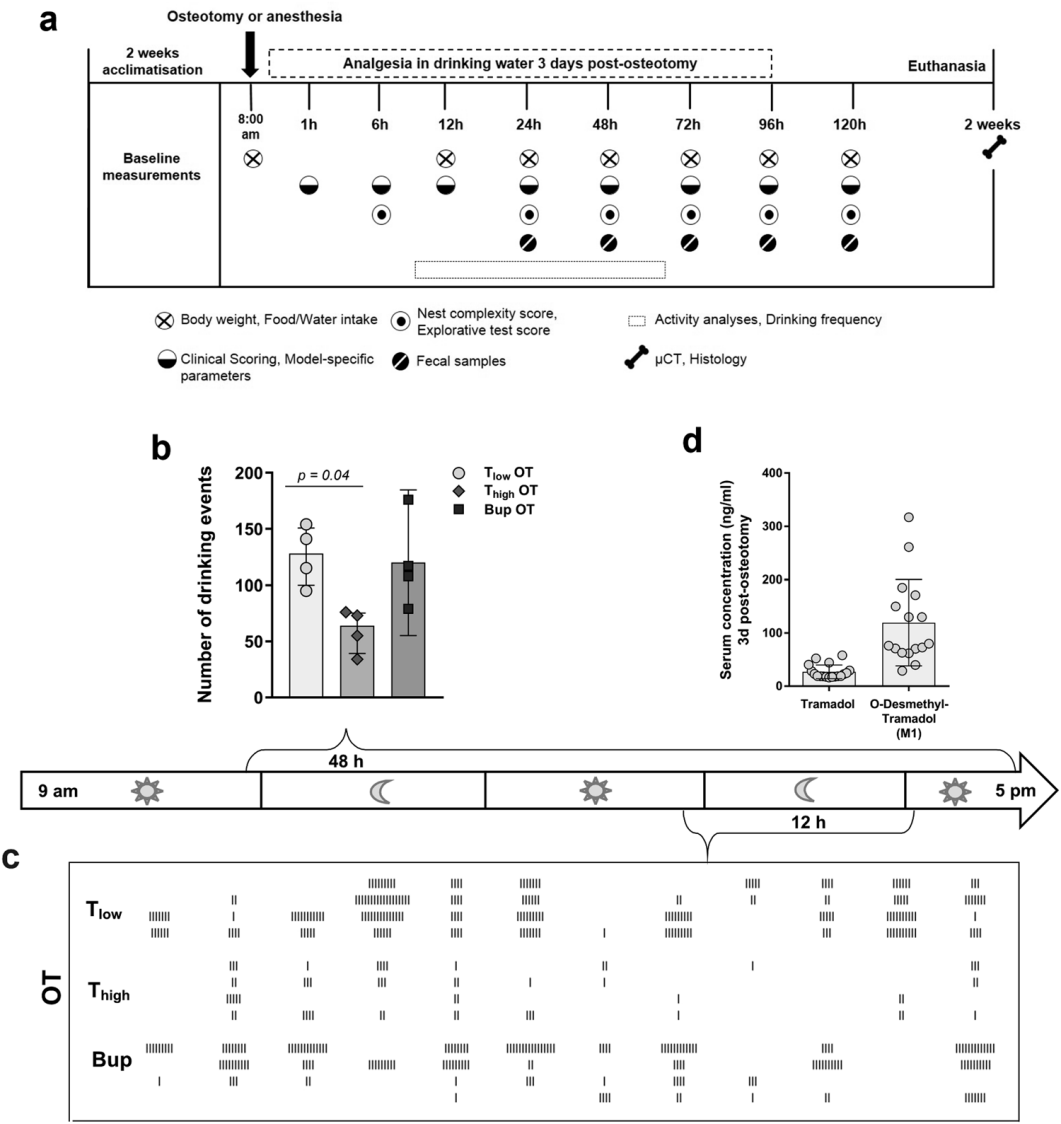
Drinking frequency is reduced concentration-dependent by Tramadol. (**a**) Study design. Overview on time points and measurements of the different parameters. (**b**) Total number of drinking events for 48 h was assessed via video recording. Data are shown for OT groups as scatter dot plot and bar with median ± interquartile ranges (n = 4). For group differences Kruskal-Wallis test and Dunn’s multiple comparison test were applied; adjusted *p*-values are indicated. Results of AN and DW groups are provided as Supplementary Fig. S1. (**c**) Depiction of the drinking events over 12 h between 36 h and 48 h post-osteotomy. Each line indicates one drinking event (n = 4). (**d**) Concentrations of Tramadol and M1 3 days post-osteotomy were analyzed in sera from mice treated with the same regime as the T_low_ OT group. Data are shown as scatter dot plot and bar with mean ± SD (n = 18).

Overall water intake was reduced compared to baseline in the T_high_ osteotomy (OT) group at 48 h and 72 h after surgery (Table 1, for other groups see Supplementary Table S1). Prior to surgery or start of treatment, the baseline of the drinking frequency over 48 h was assessed for n = 8 mice, which resulted in a median of 182 events in 48 h (interquartile range: 170.5, 207). Video analysis for 48 h starting 9 h after osteotomy enabled the examination of the drinking frequency and therefore the monitoring of continuous analgesia up-take (Fig. 1b,c). After start of the experiment, the number of drinking events decreased significantly in the T_high_ OT group compared to the T_low_ OT groups (Kruskal-Wallis test and Dunn’s multiple comparison test; H = 7.42; exact *p = *0.01; adjusted *p* = 0.04 − T_low_ vs. T_high_) and showed a non-significant tendency for reduction compared to the Bup OT (adjusted *p* > 0.99 T_low_ vs. Bup, *p* = 0.07 Bup vs. T_high_) (Fig. 1b). To evaluate the impact of the anesthesia and the treated drinking water alone on the outcome parameters, two control groups were used in the study. In the anesthesia (AN) group, animals underwent isoflurane anesthesia and were treated with either high or low dose Tramadol or Buprenorphine via drinking water for 3 days. In the drinking water (DW) group, animals received either high or low dose Tramadol or Buprenorphine via drinking water over 3 days. In the AN and DW groups, the drinking frequency was slightly higher in the Bup groups compared to the T groups (Supplementary Fig. S1). The depiction of individual drinking events indicates a decline in the drinking frequency in the T_high_ OT group between 36 h and 48 h post-osteotomy (Fig. 1c).

**Table 1:** Food and water intake per cage for the osteotomy groups.

	Food intake (g) Median (Min – Max)					Water intake (ml) Median (Min – Max)				
Groups	0 h	24 h	48 h	72 h	96 h	0 h	24 h	48 h	72 h	96 h
T_low OT_	7.69 (7.5–8.8)	5.09 (4.4–5.8)	7.92 (6.4–9.7)	7.63 (5.4–10.1)	10.25 (3.0–14.5)	9.26 (7.4–9.9)	7.26 (3.8–11.7)	9.88 (6.2–11.3)	10.67 (10.5–13.2)	7.90 (7.4–10.0)
T_high OT_	9.05 (8.5–9.2)	4.56 (2.8–14.2)	3.75 (2.9–4.0)	8.05 (5.0–9.0)	11.76 (10.2–16.8)	9.38 (8.5–11.0)	10.19 (8.7–12.5)	5.35 (3.1–11.1)	5.69 (5.0–6.3)	9.52 (9.1–9.9)
Bup OT	8.54 (8.3–9.7)	6.84 (5.2–9.8)	4.77 (4.0–6.1)	11.92 (10.1–17.1)	8.97 (5.8–12.5)	9.15 (8.2–10.2)	8.65 (4.5–11.7)	7.38 (4.5–11.6)	11.34 (10.0–12.7)	9.80 (9.4–10.9)

N = 4 cages (with 2 mice per cage). See Supplementary Table S1 for values of control groups (AN, DW).

### T_low_ Tramadol and M1 serum concentrations appear sufficient

To verify sufficient intake of Tramadol, sera from T_low,_ OT mice euthanized 3 days after osteotomy were analyzed for Tramadol and O-Desmethyl-Tramadol – the analgesic active/effective metabolite (M1) – concentrations. The mean serum concentration of Tramadol was 27.1 ng/ml (±12.9 ng/ml) and the mean M1 concentration was 119.6 ng/ml (±81.1 ng/ml; Fig. 1d).

### High concentrations of Tramadol in the drinking water impact body weight and food intake

**Figure 2:**
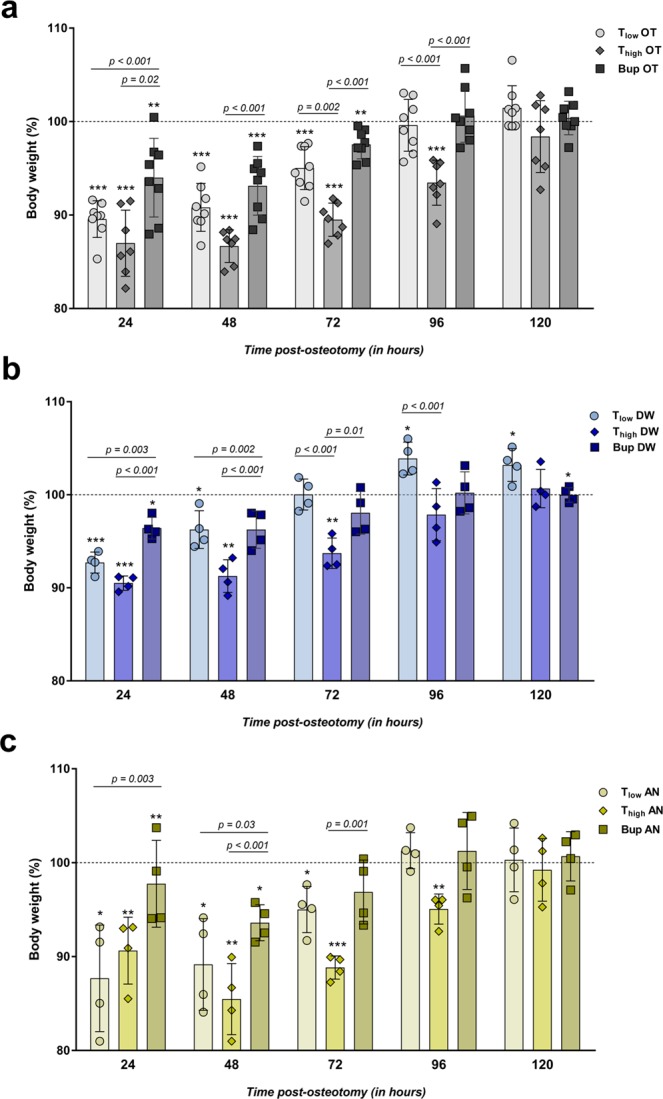
Body weight development is affected by type and concentration of opioid. (**a**–**c**) Body weight was measured every 24 h and normalized to the initial weight before surgery (=100%). Graphs depict the osteotomy groups (**a**, OT) and the control groups including anesthesia and analgesics via the drinking water (**b**, AN) and only analgesics via the drinking water (**c**, DW). Data are shown as scatter dot plot and bar with mean ± SD for n = 8 (OT) and n = 4 (AN, DW). One sample *t* test was used to determine statistical significance towards the initial body weight (hypothetical mean = 100; exact *p*-values are listed in Supplementary Table S2); *p*-values are indicated with *p < 0.05, **p < 0.01 and ***p < 0.001. For group differences One-way ANOVA with Bonferroni’s multiple comparisons test was performed; adjusted *p*-values are indicated.

As a parameter of general wellbeing, body weight was measured every 24 h and normalized to the initial body weight directly before osteotomy (baseline). Animals of all treatment groups lost weight compared to baseline values, regardless of whether they underwent surgery or not. The body weights of all groups undergoing OT were significantly reduced at 24 h after surgery compared to the initial body weight before surgery and recovered on the following days depending on the analgesia protocol (one sample *t* test; exact *p-*values are listed in Supplementary Table S2) (Fig. 2a). Bup OT and T_low_ OT mice reached initial body weight levels (≥100%) at 72 h post-osteotomy. In contrast, T_high_ OT mice showed significant decreased body weight until 120 h after osteotomy. Significant group differences were found at 24, 48, 72 and 96 h post-osteotomy, F (14, 100) = 26.21 (*p* < 0.001) (One-way ANOVA with Bonferroni’s multiple comparisons test; selected pairs comparison; adjusted *p-*values are indicated in Fig. 2a). In detail, mice of the Bup OT group revealed significant lower fold changes compared to the T_high_ OT group at 24 h, 48 h, 72 h and 96 h, respectively and the T_low_ OT at 24 h. Additionally, at 72 h and 96 h after surgery, differences were observed between the T_low_ OT and T_high_ OT groups. At 24 h after anesthesia and/or start of the analgesic treatments, the AN and DW groups showed significant lower body weights compared to the initial body weight, which paralleled the observations from the OT groups (Fig. 2b,c). Overall positive body weight development was more pronounced in the DW groups (reaching 100% − T_low_ 72 h, T_high_ 96 h, Bup 48 h) (Fig. 2b) than in the AN groups (reaching 100% − T_low_ 96 h, T_high_ 120 h, Bup 72 h) (Fig. 2c) or OT groups (reaching 100% – T_low_ 96 h, T_high_ 120 h, Bup 96 h) (Fig. 2a). There was a significant difference between the AN groups (F (14, 45) = 12.16; *p* < 0.001) and DW groups (F (14, 45) = 23.39; *p* < 0.001) at different time points (One-way ANOVA; adjusted *p-*values are indicated in Fig. 2b). In the AN groups, Bup-treated animals showed significant higher body weights than T_low_ (24 h and 48 h) and T_high_ (48 h and 72 h) treated animals. This was also observed in the DW groups (Bup vs. T_low_ or T at 24 h and 48 h; Bup vs. T_high_ at 24 h, 48 h and 72 h). Additionally, T_low_ DW groups showed significant differences towards the T_high_ DW groups at 72 h and 96 h. Intra-group differences over time were mostly significant for all OT groups and the T_low_/T_high_ AN and DW groups when applying a repeated measures two-way ANOVA with a Tukey’s multiple comparisons test (Interaction: F (40, 192) = 5.59; *p* < 0.001; Time: F (4, 192) = 130.8; *p* < 0.001; Fold change body weight – column factor: F (10, 48) = 25.84; *p* < 0.001; adjusted *p-*values for each comparison per group are listed in Supplementary Table S3).

Food intake was measured daily before and after osteotomy. All OT groups showed a reduced food intake 24 h post-osteotomy that remained low for the Bup OT and T_high_ OT groups for 48 h or 72 h, respectively (Table 1). Similar trends were observed in the AN and DW groups (Supplementary Table S1).

### Composite pain score (facial expression + appearance) decreases constantly over time in all OT groups and is strongly influenced by anesthesia and analgesia protocol

**Figure 3:**
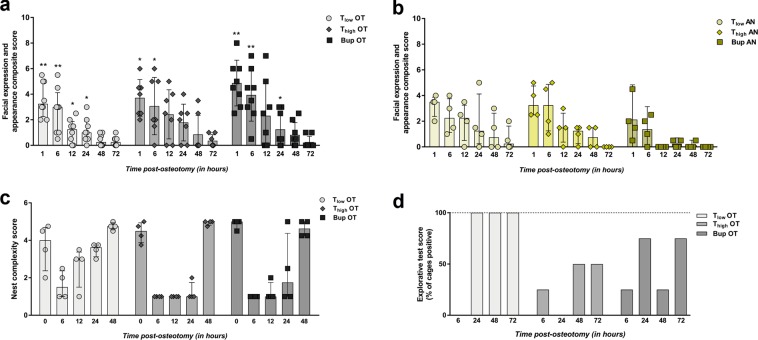
General wellbeing parameters are reduced in osteotomy groups and influenced by anesthesia and analgesia. (**a**,**b**) Mice were transferred individually to observation boxes, allowed to acclimate for 1 min and were observed for 3 min. Facial expression and appearance composite score was assessed in the OT (**a**) and AN (**b**) groups at 1, 6, 12, 24, 48 and 72 h post-osteotomy/post-anesthesia. DW groups were continuously scored with 0. Measured scores are shown as scatter dot plot and bar with median ± interquartile range for n = 8 (OT) and n = 4 (AN). Wilcoxon signed rank test was used to determine statistical significance towards the initial score (hypothetical median = 0; exact *p*-values are listed in Supplementary Table S4); *p*-values are indicated with *p < 0.05, **p < 0.01 and ***p < 0.001. (**c**) Nest complexity score of the OT groups are depicted for 0, 6, 12, 24 and 48 h post-osteotomy. Data are shown as scatter dot plot and bar with median ± interquartile range for n = 4 (cages). (**d**) The explorative test score is depicted for the OT groups as mean – percentage of cages that were determined positive for n = 4 (cages). Nest complexity and explorative test scores are provided in Supplementary Fig. S2.

A composite score consisting of parameters of facial expression and overall appearance was determined by transferring the mice individually to an observation box at 1, 6, 12, 24, 48 and 72 h post-osteotomy. In order to familiarize the mice with the procedure, scoring was performed at three time points (9 a.m., 3 p.m. and 8 p.m.) on the day before surgery or treatment (baseline). The baseline score was 0 for all mice. After surgery/treatment, OT groups showed a significant increase of the score at 1 and 6 h independent of the treatment and compared to the baseline score of 0 (Wilcoxon signed rank test; hypothetical median = 0; exact *p*-values are listed in Supplementary Table S4) (Fig. 3a). AN groups showed higher scores than baseline at several time points in the immediate post-anesthesia phase that were not statistical significant (Fig. 3b). DW groups scored continuously low as baseline (=0). Treatment group comparison (T_low_, T_high_ vs. Bup) at every time point (selected pairs comparison) using Kruskal-Wallis test (non-parametric) revealed significant differences in medians within the OT groups (H = 68.66; app. *p* < 0.001) and AN groups (H = 41.92; app. *p* < 0.001). The following Dunn’s multiple comparison test showed no significance. As shown in the graphs, the median scores reached nearly baseline values at 48 h for the T_low_ OT group and 72 h for the T_high_ OT and Bup group. This was comparable to the T_high_ AN group (72 h) while median scores of T_low_ AN reached baseline values at 72 h and Bup AN at 12 h.

### Nest building and explorative behavior are influenced by type and concentration of opioid

The nest complexity score was used to detect changes in general behavior that might indicate changes in wellbeing as described previously^22^. At 7 a.m., 1 h before surgery or treatment, the baseline nest complexity score, determined for all groups, reached almost the maximum score of 5. Normalization of the scores towards the baseline score was not performed to show the variance of the scores between the groups in absolute numbers per cage. Therefore, the baseline scores (time point = 0) are depicted within the graph (Fig. 3c). Post-osteotomy all OT groups showed a decline in the nest complexity score towards 1 (Fig. 3c). T_low_ OT groups recovered faster between 12 h and 48 h than the T_high_ OT and Bup groups comparing the medians. A statistically significant difference in group medians was found (Kruskal-Wallis test; non-parametric; H = 48.51; app. *p* < 0.001) while the Dunn’s multiple comparison test revealed no significance between the selected pairs (*p* > 0.99). However, strong differences can be seen between the T_low_ OT and T_high_ OT group at 12 h and 24 h. Animals from AN and DW groups showed a comparable decline at 6 h, but achieved the initial state more continuously and faster over the following 42 h (Supplementary Fig. S2).

Explorative behavior scores are presented in the percentage of cages that were scored positive (100% = four cages scored positive). In general, the scores were more variable in the OT groups than in the other treatment groups (Fig. 3d). While all animals showed explorative behavior in the baseline measures prior to surgery, explorative behavior declined towards zero at 6 h after surgery. T_low_ OT recovered to stable values at 24 h (=100%). Animals from T_high_ OT showed reduced explorative behavior over time compared to T_low_ OT mice. Bup OT groups demonstrated varying behavior between a mean of 25% (one cage out of four positive) and 75% (three cages out of four positive). AN groups showed comparable results to the OT groups while DW groups were highly explorative with a mean score of 100% (Supplementary Fig. S2).

Additionally, the time spent in the nest (resting time) was assessed for each individual and normalized to the total recorded time. As shown in Supplementary Fig. S2, there was no significant difference between the OT groups (Kruskal-Wallis test; H = 2.67; exact *p* = 0.28). There was a slight decrease of the relative time spent in nest with the AN and DW groups compared to the OT groups. In order to track the normal time in the nest over 48 h, baseline measurements were performed prior to surgery or treatments for n = 8 mice. The median of the relative time in nest was 47.05% (interquartile range: 43.13%, 48.18%), indicating an increase in resting time for all groups after surgery or treatments compared to normal conditions.

### Model-specific pain behavior are reduced by Buprenorphine and low concentrations of Tramadol

**Figure 4:**
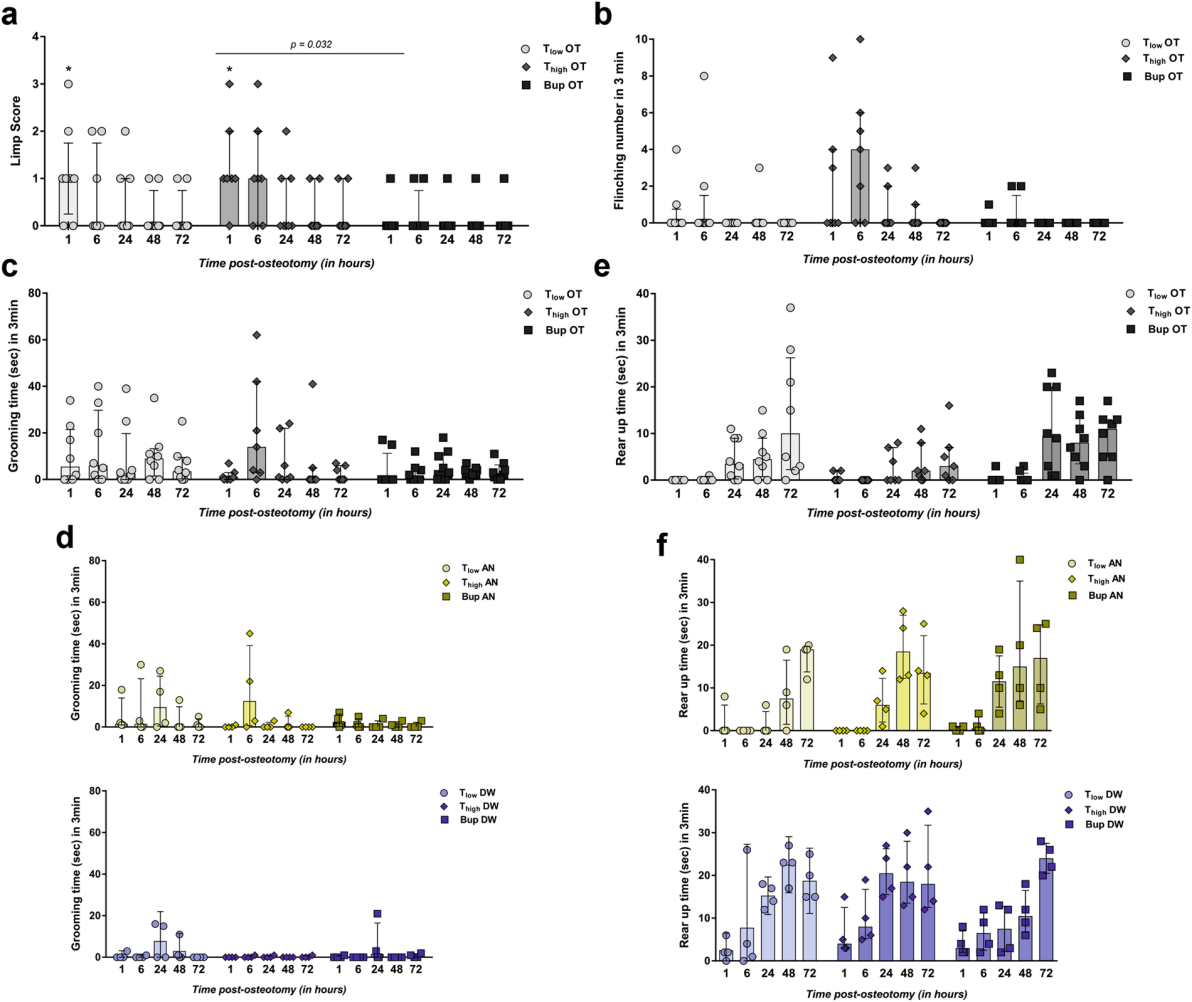
Higher concentrations of Tramadol do not further ameliorate model-specific pain-related parameters. (**a**) The limp score included limping and dragging. Only the scores for the OT groups are shown in the graph as AN and DW groups showed no positive score. Measured scores are shown as scatter dot plot and bar with median ± interquartile range for n = 8. Wilcoxon signed rank test was used to determine statistical significance towards the initial score (hypothetical median = 0; exact *p*-values are listed in Supplementary Table S5); *p*-values are indicated with *p < 0.05, **p < 0.01 and ***p < 0.001. For group differences, Kruskal-Wallis test with Dunn’s multiple comparison test for selected pairs (time point) was performed; adjusted *p*-values are indicated. (**b**) Number of flinching per 3 min observation are depicted for OT groups (no flinching was observed in AN and DW groups). (**c**,**d**) Graphs show grooming time during 3 min for OT (**c**) and AN and DW (**d**) groups. (**e**,**f**) Rear up time during 3 min is shown for OT (**e**) and AN and DW (**f**) groups. (**b**–**f**) Data are shown as scatter dot plot and bar with median ± interquartile range for n = 8 (OT) and n = 4 (AN, DW).

To assess model-specific pain behaviors, a limp score (limping and dragging), the frequency of flinching, the duration of grooming the operated leg as well as the rear up duration, i.e., time standing on both hind limbs in 3 min, were determined. Limp score and flinching are described only for OT animals as AN and DW groups showed no positive limp scores or flinching. Baseline limp score and flinching were assessed prior to osteotomy, revealing no positive limping or dragging (score = 0) and no flinching. Mice of the T_low_ OT and T_high_ OT group showed significant higher limp scores at 1 h post-osteotomy compared to the baseline scores (=0) (Wilcoxon signed rank test; hypothetical median = 0; exact *p*-values are listed in Supplementary Table S5) (Fig. 4a). Group medians were statistically significant (Kruskal-Wallis test; non-parametric; H = 29.45; app. *p* < 0.009) between the Bup OT and T_high_ OT groups (Dunn’s multiple comparison test; selected pairs comparison; *p* = 0.032). In general, the Bup OT and T_low_ OT groups showed a median score of 0 at 6 h post-osteotomy compared to the T_high_ OT group with a median of 1 that was reduced towards the baseline at 12 h post-osteotomy. Flinching was not statistically significantly increased in the OT groups compared to the baseline (=0) (Wilcoxon signed rank test; hypothetical median = 0; *p* > 0.25 except T_high_ OT at 6 h *p* = 0.063) (Fig. 4b). The Kruskal-Wallis test showed a significant difference in the group medians (H = 32.28; app. *p* < 0.004) while the Dunn’s multiple comparison test revealed no significance between the selected pairs (*p* > 0.99; except Bup OT vs. T_high_ OT *p* = 0.1 and T_low_ OT vs. Bup OT *p* = 0.15). Nevertheless, flinching was observed more often in the T_high_ OT group (median = 4) compared to the T_low_ OT and Bup OT groups (median both = 0) at 6 h (Fig. 4b).

Baseline measurements of leg grooming duration showed durations between 0 and 4 sec (n = 8); no statistical test was performed for group comparisons. However, grooming time was prolonged in OT groups for up to 3 days after osteotomy, with no difference between the three groups (Kruskal-Wallis test; non-parametric; H = 15.28; app. *p* = 0.36) (Fig. 4c). Interestingly, increased grooming was also seen in T_high_ AN, T_low_ AN and T_low_ DW mice (Fig. 4d).

Normal rear up time was determined during baseline measurements (n = 8) with a median = 14.2 sec and a range from 8 to 27 sec. No statistical test was performed for comparison with baseline. Rear up was shown only rarely by OT groups at 1 h and 6 h post-osteotomy (Fig. 4e), which is in line with the results of the AN groups (Fig. 4f). Between 24 h and 96 h, rear up time increased in the OT as well as in the AN and DW groups (Fig. 4f). Statistical differences in the OT group medians were found (Kruskal-Wallis test; non-parametric; H = 60.03; app. *p* < 0.001) while Dunn’s multiple comparison test revealed no significance between the selected pairs (*p* > 0.99; except Bup OT vs. T_high_ OT *p* = 0.36). In general, rear up was more apparent in the Bup OT and T_low_ OT compared to the T_high_ OT group.

### Physiological stress response varies in regard to osteotomy surgery and analgesic treatment

**Figure 5:**
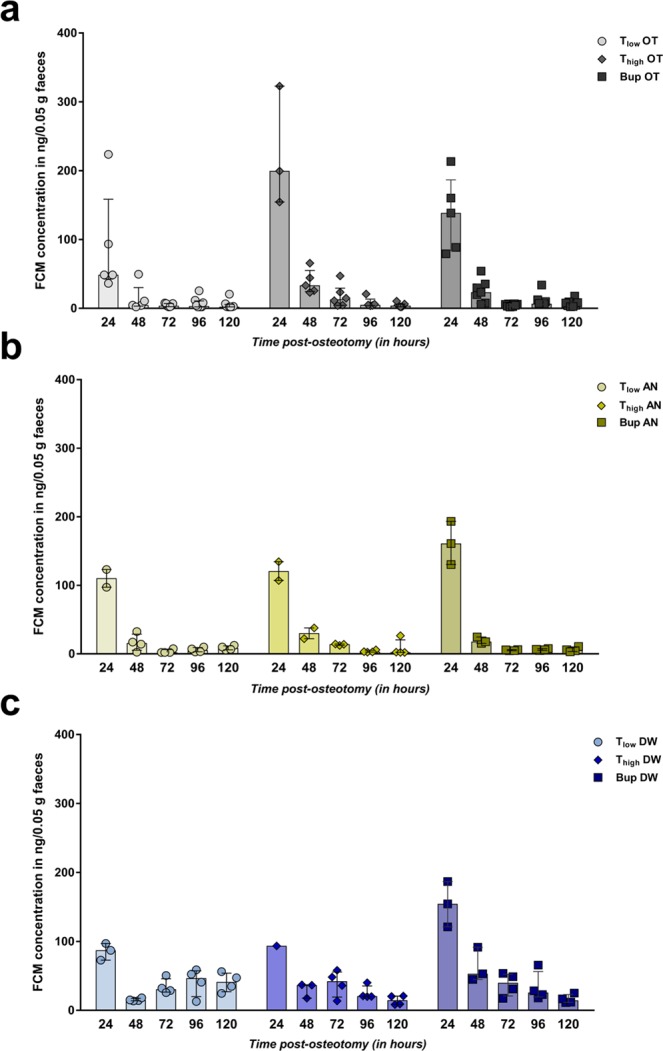
Assessment of adrenocortical activity indicate varying physiological stress responses. (**a**–**c**) Fecal corticosterone metabolite (FCM) concentrations were analyzed in all groups: OT (**a**), AN (**b**) and DW (**c**) group. Data are shown as scatter dot plot and bar with median ± interquartile range for n = 8 (OT) and n = 4 (AN, DW).

To evaluate stress non-invasively, FCMs were determined. Samples were collected during the animals’ stay in the observation boxes after spontaneous and voluntary defecation (no samples at 1 h, 6 h and 12 h post-osteotomy/post-anesthesia). Consequently, different numbers of fecal samples were collected per time point and group. FCM concentration peaked at 24 h post-osteotomy in the OT groups, with no significant differences between groups (Kruskal-Wallis test; H = 3.82; *p* = 0.15; Fig. 5a) and declined immediately at 48 h to values lower than DW mice (Fig. 5c). Nevertheless, FCM concentrations in AN and DW groups were also slightly elevated at 24 h and declined over time (Fig. 5b,c).

### Opioid analgesia does not significantly affect fracture healing outcome

**Figure 6:**
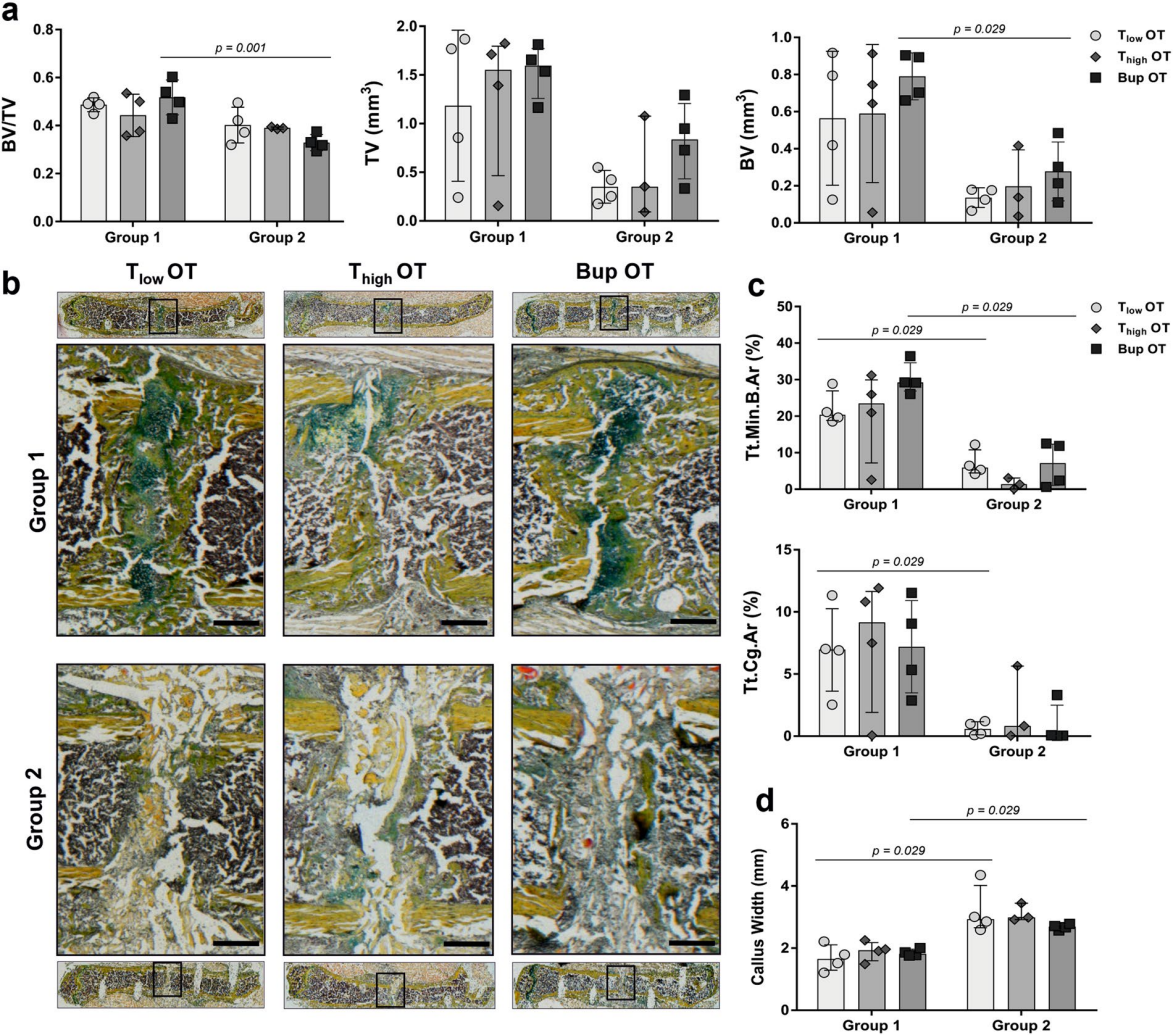
Fracture healing outcomes are not affected by type and concentration of opioid. (**a**) Using *ex vivo* µCT, the bone volume (BV), the total volume (TV) and the bone volume fraction (BV/TV) were determined. (**b**) Exemplary images for Movat’s pentachrome staining allowing for the differentiation between tissues: yellow/orange = mineralized bone; light yellow = scaffold; green = cartilage; purple/brown = bone marrow and cells. Scale bars = 500 µm. (**c**) Histomorphometry was performed for quantitative assessment of different tissue components – mineralized bone (Tt.Min.B.Ar), cartilage (Tt.Cg.Ar) and (**d**) callus width. (**a**–**d**) Data are shown as scatter dot plot and bar with median ± interquartile range for n = 3–4. Differences between group 1 and 2 within one analgesic group were determined using the Mann-Whitney U test. Exact *p*-values are indicated in the graphs.

In order to evaluate the potential side effects of opioid pain management on fracture healing outcomes, we performed *ex vivo* µCT, histomorphometric analysis and vessel staining via immunofluorescence. Within the protocol, animals from each OT group were assigned randomly to one of two groups receiving different treatment of the osteotomy gap. While in group 1, the osteotomy gap was empty, a scaffold was applied in group 2 that inhibits fracture healing, as we have shown previously^21^. In the present study, we focused on potential differences within group 1 or 2 to evaluate the potential influence of the pain management regime. The total volume (TV), bone volume (BV) and bone volume fraction (BV/TV) were determined during *ex vivo* µCT analyses. The BV revealed no significant differences between the analgesics groups within the treatment groups (Kruskal-Wallis test; selected pairs; H = 10.01; *p* = 0.75) while the medians of TV and BV/TV were significant (Kruskal-Wallis test; selected pairs; TV: H = 11.18; *p* = 0.05; BV/TV: H = 13.05; *p* = 0.02), but not in the Dunn’s multiple comparison test (*p* > 0.99) (Fig. 6a). Moreover, we detected a significant difference in the Bup OT group between group 1 and 2 (Mann-Whitney U test; *p*-values indicated in graphs) (Fig. 6a). Histomorphometric results analyzing the total mineralized bone (Tt.Md.B.Ar) and cartilage area (Tt.Cg.Ar) as well as the callus width were significantly different in group medians within one fracture gap treatment group and between the analgesic protocols (Kruskal-Wallis test; selected pairs; Tt.Md.B.Ar: H = 16; *p* = 0.007; Tt.Cg.Ar: H = 11.05; *p* = 0.05; Callus width: H = 17.89; *p* = 0.003). Dunn’s multiple comparison test showed no significance (*p* > 0.99) (Fig. 6b–d). Within group 1, Bup OT revealed a higher Tt.Md.B.Ar compared to the T OT groups, but conversely, T_high_ OT exhibits slightly more cartilage within the fracture gap. Between the treatment groups, significant differences were found for T_low_ OT and Bup OT, showing significant higher amounts of mineralized bone and cartilage in group 1 compared to group 2 (Mann-Whitney U test; *p*-values indicated in graphs) (Fig. 6c). This was also observed for the callus width, which was also significant higher in the T_high_ OT group 2 compared to group 1 (Fig. 6d).

**Figure 7:**
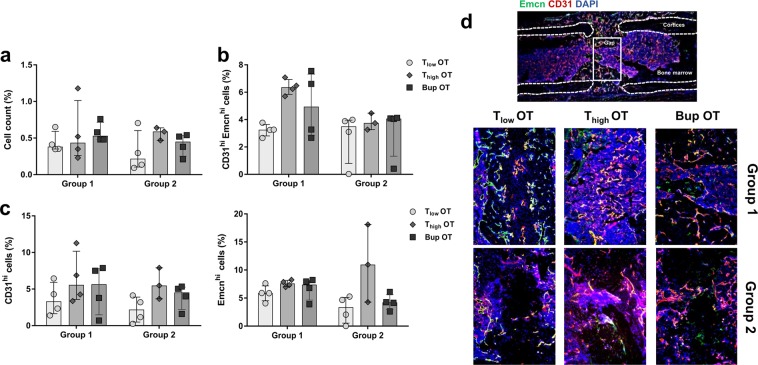
Vessel formation is comparable between treatment groups. Immunofluorescence staining was performed to measure (**a**) the cell count, (**b**) the number of double-positive cells (CD31^hi^ Emcn^hi^) and (**c**) the number of CD31^hi^ and Emcn^hi^ cells alone in the osteotomy gap. Data was normalized to the total analyzed area and are shown as scatter dot plot and bar with median ± interquartile range n = 3–4. (**d**) Exemplary immunofluorescence vessel staining (CD31 = red, Emcn = green, DAPI = blue). Scale bars = 200 µm.

To evaluate vessel formation within the fracture gap, staining was performed for CD31 and endomucin (Emcn), two common endothelial markers. In a first step, the relative cell count was determined within the fracture gap (Fig. 7a). No significant differences were identified between analgesic protocols or treatment groups (Kruskal-Wallis test; H = 4.65; *p* = 0.46). This was also observed for CD31 and Emcn alone (Kruskal-Wallis test; CD31: H = 6; *p* = 0.31; Emcn: H = 10.85; *p *= 0.054) (Fig. 7c). Therefore, the obvious higher amount of Emcn^hi^ cells in the T_high_ OT group 2 seems to be an effect of random sampling. Additionally, there were no differences between treatment groups for CD31^hi^ Emcn^hi^ (Kruskal-Wallis test; H = 9.54; *p* = 0.089) although the Dunn’s multiple comparison test showed a significance in group 1 between the T groups (*p* = 0.04) (Fig. 7b).

### Unexpected observations of the study

**Figure 8:**
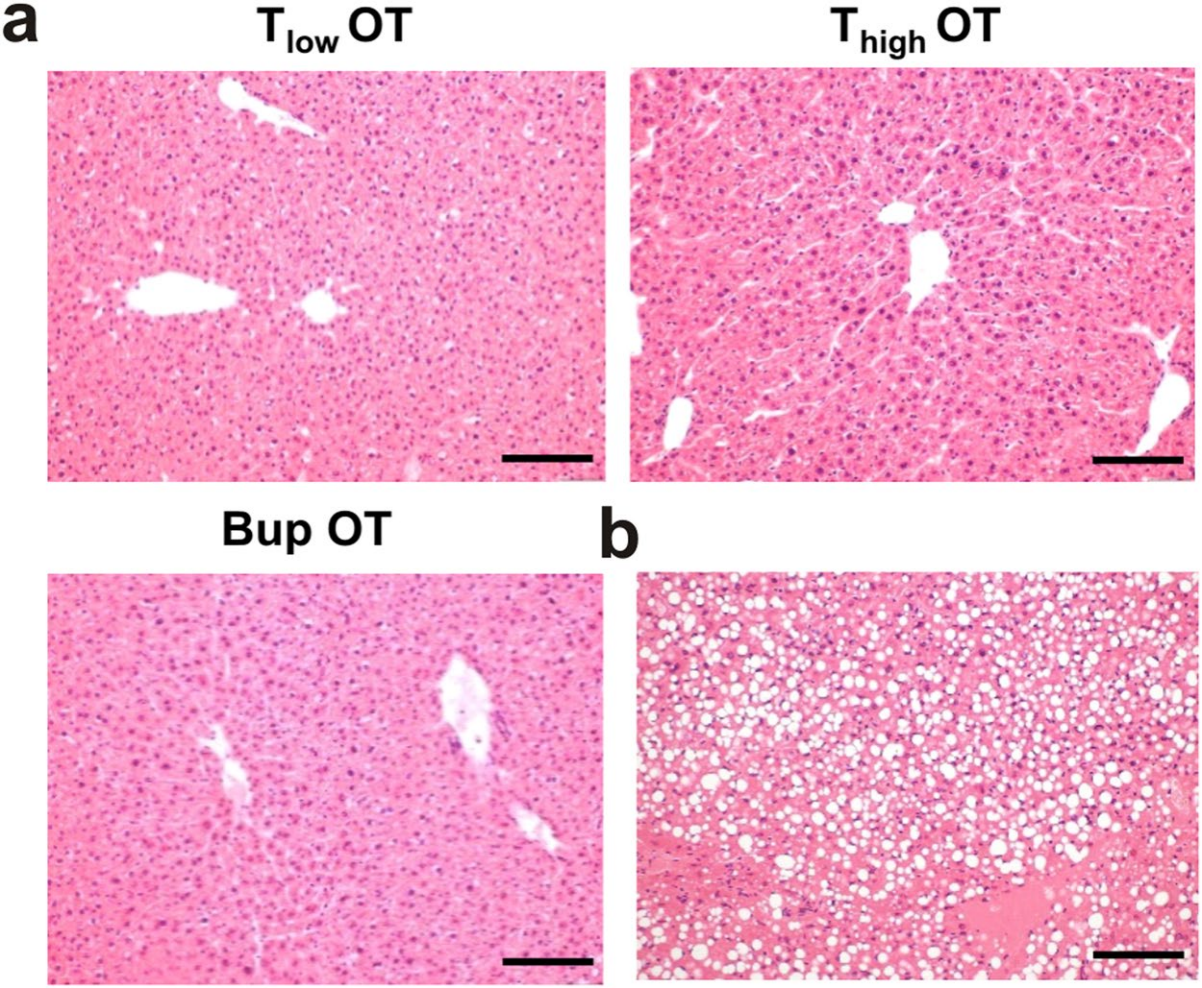
Liver histology reveal no differences between groups. (**a**) Histology of the livers from all OT animals (n = 4) was performed as one animal needed to be euthanized 48 h after osteotomy; this animal showed a high-grade fatty liver (**b**). Scale bars = 200 µm.

One animal from the T_high_ OT group was euthanized 2 days after osteotomy due to reaching a humane endpoint. Autopsy revealed a hepatomegaly. In order to exclude the influence of the pain medication, this liver as well as the livers of all other animals from the OT group were assessed via histology; the results revealed a high-grade fatty liver in the respective animal (Fig. 8b) and no changes in the others (beside mild hepatitis due to *Helicobacter* infection) (Fig. 8a).

## Discussion

This study evaluated three used analgesia protocols, Tramadol (0.1 mg/ml = T_low_ vs. 1 mg/ml = T_high_) and Buprenorphine (0.009 mg/ml) administered via drinking water, in a mouse osteotomy model after a pre-operative injection of Buprenorphine. Analgesia, anesthesia, surgery and testing had clear effects on the measured parameters assessed. All protocols tested reduced pain without side effects on fracture healing outcome. Nevertheless, mice that were treated with T_high_ showed more signs of reduced wellbeing and pain compared to mice treated with T_low_ or Buprenorphine.

Daily water intake in female B6 mice is about 3–6 ml^23^, which resembles our baseline values. After osteotomy water intake was reduced but remained sufficient for adequate analgesia in groups receiving Buprenorphine or T_low_. In contrast, water intake and drinking frequency were reduced significantly in animals that received T_high_ after surgery. While overall, water intake was not distinctly reduced in controls without surgery receiving T_high_ compared to other analgesia protocols, drinking frequency during the first 2 days was low compared to animals that received a lower dose or Buprenorphine. Comparable effects were observed in a previous study: in contrast to sufficient overall 24 h intake of Tramadol-treated water after laparotomy in female B6 mice, drinking frequency was distinctly reduced in the acute post-surgical phase (<4 h)^24^. Reduced water intake might result in insufficient concentrations of Tramadol or M1, the analgesic metabolite of Tramadol, and, therefore, in insufficient pain relief. However, based the amount of water intake, the total amount of Tramadol intake can be calculated and compared between the T_low_ and T_high_ groups. Considering a total water up-take at 48 h for two mice with a median of 9.88 ml (T_low_) and 5.35 ml (T_high_), the calculated up-take of Tramadol per mouse would be 0.49 mg (T_low_) compared to 2.68 mg (T_high_) which is a 5-fold difference and indicates a sufficient up-take of Tramadol in T_high_ although the water intake amount was reduced. Nevertheless, this calculation does not include the timing of the water intake over 24 h and the short half-life of Tramadol^14^. It can be assumed that the bitter taste or aversive side effects of T_high_ result in reduced willingness to ingest. As effective drinking water administration relies on frequent and sufficient water intake, sweetened drinking water as well as application of pain medications via the drinking water prior to surgery might be used to avoid the observed effect and to ensure sufficient intake^16,25^. In addition, the voluntary ingestion via nut paste, jelly^26^ or by syringe feeding can be an alternative in order to ensure the adequate intake, although animals should be used to the food or technique beforehand^27^.

We determined the serum levels of Tramadol and M1 following administration of T_low_. The M1 value was found to be nearly three times as high than the human minimal effective serum level of M1^28^. Nevertheless, analgesic serum concentrations of Tramadol or M1 in mice are not known; we can therefore only assume that mice received sufficient doses.

Weight reduction, and reduced food intake, might be indicators of pain^10,29^ but are also known side effects of analgesics^10^. All animals lost weight after the start of experiments, suggesting an effect of treatment but also of the testing procedure. This tendency was stronger after osteotomy and anesthesia – a result that can possibly be explained by a loss of fluid during anesthesia and the observed drop in food and water intake after anesthesia. The impact of surgery on these parameters was minor compared to the effect of analgesia and anesthesia only. Interestingly, animals treated with T_high_ lost more body weight and recovered weight more slowly compared to the other groups. This effect was seen in surgery and controls, and can therefore be more likely attributed to the side effects, of T_high_ and less likely to pain due to osteotomy.

Composite scores comparable to the scoring system used, have been applied successfully for pain evaluation in other mouse models (e.g.^10,30^). In our study, scores increased during the acute post-anesthesia and post-osteotomy phase. Nevertheless, the increase in scores towards mean scores of 3–6 was significant compared to baseline in the surgery groups only and scores of osteotomized animals recovered slower towards baseline levels compared with anesthesia groups. In a previous study, we showed that, after ovariectomy, B6 mice that did not receive pain treatment reached mean composite scores of 4.5, while animals that received effective pain treatment after surgery reached mean scores of 1.5–2.5^10^. Compared to the increase in scores after anesthesia and analgesia without surgery, osteotomized animals showed only slightly higher scores, hinting at very mild residual post-surgical pain.

The influence of anesthesia is in line with findings that subunits of the score are not pain-specific. An increase in the MGS, part of the pain scoring used in our study, after anesthesia is also described by Miller *et al*.^31^.

High nest scores – an indicator of good general wellbeing^30,32,33^ – were observed during baseline. Nest scores were reduced in all groups after the start of experiments. This reduction in nest building is a known side effect of anesthesia and opioid analgesia^32,34^. Nevertheless, the impact on nest building was less severe in animals that received T_low_ compared to T_high_ or Buprenorphine, hinting at fewer side effects on wellbeing. Changes in activity might have been the underlying cause of reduced nest building. Groups that scored higher in nest scores in general also spent less time inactive in their nest.

The explorative test used in our study is a modification of the time-to-integrate-to-nest test (TINT)^35^. Mice observed in pilot studies rarely started to integrate provided nest building material into their existing nest within 10 min as described by Rock *et al*., therefore we assessed exploration of new nest material rather than nest building behavior. Exploration might be a useful measure of activity and anxiety^36^ and therefore an interesting parameter to assess overall wellbeing in mice. In the explorative test, all mice scored positive in baseline, animals that received analgesia only showed a comparable high level of explorative behavior during the observation time. After anesthesia, exploration was reduced in all OT groups. This reduction seemed to be stronger in animals that received T_high_, suggesting a negative effect of the higher dose on wellbeing.

Assessment of limp scores and rearing behavior to quantify altered gait and weight bearing are commonly used in bone fracture, neuropathy or cancer pain studies^37–40^. Additionally, grooming in terms of an increased tending to the painful limb and flinching as a pain-specific symptom is applied^41–43^. No limping, flinching or guarding was observed during baseline or in the AN and DW groups. Limping and flinching occurred rarely after osteotomy. Rear up time was shorter in surgery groups compared to AN and DW groups. These changes are therefore clearly connected to the surgery and do not represent a side effect of analgesia or anesthesia.

We cannot discriminate if these changes are due to functional impairment, due to the fixator, or residual osteotomy pain. Minville *et al*. reported that mice with a fractured tibia have a significantly higher “subjective pain” score, defined as reduced use of fractured limb with regards to limping and weight bearing, than sham-operated animals^40^. This score was reduced with an opioid + NSAID combination, which proves the pain specificity of the parameters. In other studies using fractured femurs, flinching occurs more frequently than in our model when animals are not treated with analgesia^42,43^. In contrast to our model, these studies used an internal pin to fixate the closed fracture.

Regardless of dose, animals that received Tramadol showed higher limp scores and more flinching than animals that received Buprenorphine. T_high_ animals showed a tendency towards more limping and flinching and less rearing and a slower recovery of behaviors. Buprenorphine treatment seemed to be slightly superior to Tramadol.

Guarding behavior, even though observed typically in rodent bone fracture pain models^41–44^, was not observed in our mice. Again, the lack of could therefore be a sign of sufficient or acceptable pain relief. Grooming of the wounded leg, which might be a sign of pain^45^, occurred in all animals, but was increased mainly in the operated and anesthetized animals. As both groups received a dressing spray to close the wound, it is assumed that grooming is not due to pain but rather induced by the sticking spray on the animal’s skin.

Measuring FCM is a non-invasive technique to monitor adrenocortical activity^46–49^. At 24 h after start of the experiments, elevated FCM levels were observed with no significant differences between groups. The increase in FCM concentrations in AN and DW groups on day one could be explained by frequent handling, which has been shown to potentially induce an acute stress response in mice^50,51^.

Finally, we evaluated the influence of Tramadol and Buprenorphine on fracture healing outcomes. No differences were found between analgesic groups in formation of mineralized bone, cartilage and vessels. In addition, we reproduce our previous finding on applying a scaffold to inhibit fracture healing^21^. Negative effects of opioids on bone homeostasis in humans have been described only in case of long-term administration, and include a high incidence of spontaneous fractures related to, e.g., loss of bone mineral density due to direct effects on osteoclasts and osteoblasts or indirect effects via the endocrine system^52–54^. However, results from animal experiments do not clearly indicate any influence of either Tramadol or Buprenorphine on bone homeostasis and/or regeneration^55,56^.

Within the study only female B6 mice were used without determination of the estrous cycle status. Therefore, we are not able to exclude potential hormonal influences, which have been also shown to be strain-dependent^57^. In addition, conclusion towards male mice should be carefully taken. Moreover, the presented analgesia protocols could be further refined by sweetening the medicated drinking water for better water intake as well as the use of local anesthesia during surgery.

Although we cannot rule out minor, short-lasting pain in the post-surgery phase, this study provides evidence for pain relief after osteotomy in female B6 mice when administering analgesics stress-free and continuously via the drinking water. Additionally, potential overdosing of Tramadol can lead to changes in wellbeing without further pain relief. Nevertheless, as we have not compared oral with injection protocols, we cannot evaluate if the injection of analgesics or the combination of injection and and oral administration for several post-surgical days might have been superior, i.e. reduces remaining signs of pain further, compared to the oral protocols. Therefore, further model-specific studies on pain management should be performed to provide evidence-based refinement of analgesia protocols, to enhance animal wellbeing, reproducibility and translation.

## Methods

### Animals, housing and study design

The study was performed in accordance with the German Animal Welfare Act and was approved by the local Berlin state authority – “Landesamt für Gesundheit und Soziales” (LaGeSo; permit numbers: G0039/16 and G0111/13). A total of 48 female C57BL/6 N mice aged 10 weeks was purchased from Charles River Laboratories (Sulzfeld, Germany). Only female mice are used routinely in our osteotomy model as bone healing is slower in female mice than in males, allowing for a stepwise, complete bone healing within a time period of 21 days^27,58,59^. The mean body weight was 21.3 ± 1.3 g. Mice were housed in a conventional (non-SPF) facility, habituated to the housing room in groups of 8–10 animals for 1 week in Eurostandard Type lll clear-transparent plastic cages and afterwards randomly split into groups of two for another week before experiments started. Mice were housed in Eurostandard Type II clear-transparent plastic cages (two animals per cage) covered with a wire lid and built-in u-shaped feed hopper and closed with a filter top. As bedding material, fine wood chips (Lignocel FS 14, J. Rettenmaier & Söhne GmbH + Co. KG, Germany) and nesting material (Envirodri®, Shepherd Specialty Papers, USA) was provided. Due to the external fixator, houses and pipes are contraindicated in order to avoid injuries. Food (Standard mouse diet, Ssniff Spezialdiäten, Germany) and tap water was available *ad libitum*, and room temperature was constant, at between 20 and 22 °C with a humidity of 45–50%. The light/dark cycle was a 12/12-h cycle with lights on at 0600 hours and off at 1800 hours. Cage changing was performed weekly during the acclimatisation phase (two weeks prior to start) by the experimenter; animals were tail handled. Nesting material was transferred to new cages when dry and clean. All experimenters were female (see^60^).

The study design (Fig. 1a) began with baseline measurements during the week prior to the osteotomy. Cages were allocated randomly to three post-operative treatment groups: Tramadol (Drops, Grünenthal GmbH) was applied in two different concentrations: T_low_ = 0.1 mg/ml and T_high_ = 1 mg/ml, Buprenorphine (Temgesic, RB Pharmaceuticals Limited) was applied with Bup = 0.009 mg/ml. Analgesics were administered via drinking water (tap water) for three consecutive days directly after osteotomy or anesthesia. The medicated water was not changed during treatment time^13^. Eight animals of each analgesic regime were osteotomized (OT), four animals of each regime underwent anesthesia only for 15 min and received analgesics post-anesthesia (AN) and four animals of each regime received only analgesics (DW; Supplementary Table S6). Two weeks after surgery, osteotomized animals were anesthetized with ketamine and xylazine (i.p. ketamine 120 mg/kg, xylazine 16 mg/kg). Once depth of anesthesia was achieved, blood was withdrawn from the heart and euthanasia was conducted by cervical dislocation.

### Osteotomy

All animals received Buprenorphine (0.03 mg/kg) s.c. as analgesic 1 h prior to surgery. Anesthesia was induced at 2.5% isoflurane (CP-Pharma, Germany) in a transparent Plexiglas box where anesthetic gas was provided using a rodent inhalation anesthesia system (Ohmeda Isotec 4, DRE, USA). Once the animal was asleep and breathing independently deeply and continuously, it was moved to a heating mat (37 °C) and anesthesia was maintained via a nose mask at 1.5% isoflurane. Eye ointment and clindamycin (0.02 ml) were applied s.c. After shaving and disinfecting the left femur area with an alcoholic iodine solution (Braunoderm, B. Braun, Germany), osteotomy was performed as described earlier^20^. In short, a lateral longitudinal incision of the skin (2 mm) along an imaginary line from knee to hip followed by a blunt preparation of the femur between the *Musculus vastus lateralis* and *Musculus biceps femoris* while protecting the subjacent nerve. Pin placement (0.45 mm diameter) was performed through the connector bar of the external fixator (MouseExFix, RISystem, Davos, Switzerland) by serially drilling to place the fixator laterally parallel to the femur. A 0.70-mm osteotomy gap was created between the middle pins using a Gigli wire saw (RISystem, Davos, Switzerland). According to the protocol of the basic research study, the osteotomy gap was either flushed with NaCl and left empty for group 1 or filled with a PBS-soaked bovine collagen-I scaffold, Lyostypt® for group 2 (B. Braun Vet Care GmbH, Tuttlingen, Germany). After skin closure, the wound was covered with a permeable spray dressing, the mice were given pre-warmed NaCl (0.2 ml) s.c. and then returned to their cages. Recovery from anesthesia was conducted under an infrared radiator and was monitored closely. Surgery was performed by two trained veterinarians. Approximately 45 min after surgery, cages were transferred back to the housing room for further analgesic treatment and behavioral assessment. General scoring and humane endpoints were applied as recommended and summarized in Lang *et al*.^27^.

### Body weight, behavioral home cage analyses and food/water intake

Animals were weighed daily starting 1 day prior to surgery and continuing until the day of euthanasia. Food and water intake were determined for each cage every 12 h. For each analgesic treatment group, 8 animals (4 OT, 2 AN, 2 DW) were filmed in their home cages starting for 48 h at 6 p.m. (9 h after osteotomy). This time point was chosen as we expected the pre-emptive Buprenorphine treatment to deliver a pain relief for up to 8 h. Cages were filmed from above (at a distance of ~ 1.5 m) with an infrared sensitive USB 3.0 Monochrome-Camera (The Imaging Source Europe GmbH, Germany). Cage grids were removed for better visibility and plastic walls elevated to prevent escape. Mice were kept with their familiar partner, provided with nesting material, food and water. One mouse in every cage was marked with black stripes (Edding permanent marker 3000) on the tail. An infrared light was turned on during dark cycles. Videos were analyzed manually by one blinded observer with event-logging software (BORIS - Behavioral Observation Research Interactive Software). The drinking frequency of each mouse was assessed manually, where the contact of a mouse’s snout with the bottle tip was counted as one drinking event and continuous drinking for a longer time was also counted as one drinking event. Time spent active was measured (defined as animal moving outside of the nest) and used to calculate resting time in the nest.

### Tramadol/M1 analysis in serum

There is no available data on the minimum effective serum concentration of Tramadol in mice; Evangelista *et al*. only recently published data on the pharmacokinetics of Tramadol with different application routes^13^. Due to this lack of knowledge, sera were additionally collected and analyzed during an unrelated parallel study in our lab. Serum from mice (n = 18) euthanized 3 days after osteotomy and treated with Tramadol via drinking water at a concentration of 0.1 mg/ml (comparable to analgesic regime of the T_low_ group) were analyzed by TOXILAB Ludwigsburg for Tramadol and O-Desmethyl-Tramadol (M1) concentrations. The analysis was conducted based on an established method to determine the concentration of Tramadol and M1 in urine. Inter- and intra-assay controls and QM were applied.

### Clinical, behavioral and model-specific pain scoring

Animal scoring was performed by two researchers blinded to the groups’ analgesic treatment. To assess the facial expression and appearance composite score, mice were transferred individually to a transparent plastic observation box, allowed to acclimate for 1 min and were observed for 3 min. Scoring was performed according to Jirkof *et al*.^61^ but slightly adapted (Supplementary Table S7). A maximum score of 11 could be reached. The nest complexity score was assessed in the home cage using the naturalistic system developed by Hess *et al*.^22^. To assess motivation to interact with an object in the home cage, we performed an explorative test. A ball of four Envirodri® stripes was put in the home cage and the animals were observed for 1 min by a blinded observer. The outcome was either negative (score = 0) with no interaction or positive (score = 1) if there was an interaction with the provided material. Interaction was defined as gnawing, fraying, carrying, holding with forelimbs, rolling and intensive sniffing of the material. As model-specific pain or severity parameters, several established behaviors related to gait and weight bearing were observed^40,43,62^. Mice were transferred individually in standard cages covered with a filter top and given an acclimation time of 15 min before taking a video for 3 min. Offline video analysis was performed by one blinded observer. The frequency of limping and dragging with the operated leg (=limp score) as well as total time an animal spent with grooming the operated leg was measured in seconds (see Supplementary Table S8). Additionally, the frequency of flinching with the left hind limb was recorded. Flinching is characterized as a rapid, repetitive lifting of the affected limb^40^. The total time of remaining in a rear up position with weight bearing apparently on both legs was determined in seconds.

### Measurement of fecal corticosterone metabolites (FCMs)

Following observation, fecal samples were collected from the observation box, and urine was carefully absorbed with a facial tissue. Fecal samples were stored at −20 °C immediately after collecting. FCM analysis was carried out as described and validated previously^46,63^. In short, the material was dried at 70 °C, homogenized with a mortar and weighed. A portion of 0.05 mg of each sample was vortexed for 2 min in 1 ml 80% methanol followed by a centrifugation step (2,500 × g for 15 min). Supernatants were stored at −80 °C and analyzed using a 5α-pregnane-3β,11β,21-triol-20-one enzyme immunoassay^63^.

### *Ex vivo* µCT of osteotomized femora

Three-dimensional (3D) bone formation was analyzed after euthanasia using high-resolution µCT. Osteotomized femora were fixed in 4% PFA and ascending glucose solutions. After removal of the pins and external fixator, femora were scanned with an isotropic voxel size of 10.5 μm (Viva40 micro-CT, Scanco Medical AG®, Switzerland, 70 KVp, 114 μA). The scan axis coincided with the diaphyseal axis of the femora while 191 slides were scanned between the middle pins to include the complete osteotomy gap. The provided software package (Scanco®, Switzerland) was used for 3D reconstruction, post-processing and analyses. The osteotomy gap was defined per sample from half broken up cortical bone to the other before the volume of interest (VOI) was defined manually excluding the cortical bone. A fixed global threshold of 240 mg HA/cm^3^ was applied for the automatic 3D callus tissue analysis. The total volume (TV, mm^3^), the total bone volume (BV, mm^3^) and the bone volume fraction (BV/TV) were included for evaluation. Analysis was performed blinded to treatment.

### Histology and immunofluorescence of osteotomized femora

In order to investigate undecalcified bones, embedding and slice preparation was conducted as described previously^21^. In detail, femora were fixed for 6 h in 4% PFA followed by an ascending sugar solution treatment (10%, 20%, 30%) for 24 h, respectively, and cryo-embedding with SCEM medium (Sectionlab, Japan) and subsequent storing at −80 °C. A specific cryofilm (Sectionlab, Japan) was used to provide slices (7 mm) with a cryotom and storage at −80 °C. Slices were air-dried for 20 min and fixed with 4% PFA prior to every histological staining. Movat’s Pentachrome staining was conducted using an already published protocol^20^. Pictures were taken with a LSM 710 confocal microscope (Carl Zeiss, Jena, Germany) and analysed using ImageJ. For immunofluorescence staining, the slides were air dried for 20 min, rehydrated with PBS and blocked with PBS/5% FCS for 30 min. Primary PECAM-1 antibody (goat anti-mouse; R&D Systems; AF3628) was diluted 1:50 in PBS/5% FCS/0.1% Tween 20 and incubated for 2 h. After washing with PBS/0.1% Tween 20, secondary antibody (donkey anti-goat A568; Life Technologies/ThermoScientific; A-11057) was diluted 1:500 in PBS/5% FCS/0.1% Tween 20 and applied for 1 h. A subsequent washing step was followed by a blocking step with PBS/10% normal goat serum. Primary Endomucin antibody (rat anti-mouse Endomucin; Santa Cruz; V.7C7) diluted 1:500 in PBS/5% FCS/0.1% Tween 20 was applied for 2 h. After washing secondary antibody (goat anti-rat A647; Life Technologies/ThermoScientific; A-21247) was used for 1 h. A washing step and DAPI (1:500; 1 mg/ml in PBS) completed the staining. Pictures were taken with a Keyence fluorescence microscope BZ 9000 using the DAPI, TexasRed and Cy5 channels. Image analysis was performed using ImageJ by a treatment-blinded researcher.

### Liver histology

The liver of each osteotomized mouse was collected after euthanasia, frozen, and stored at −80 °C. Before embedding in paraffin, the livers were fixed in 4% PFA for 24 h and then transferred to the Institute of Veterinary Pathology at the Department of Veterinary Medicine FU Berlin. The livers were paraffin embedded and HE stained. The samples were assessed histologically blinded to the analgesic treatment to assess any possible influence of the analgesia on the liver.

### Statistical analysis

Samples size calculation for primary endpoints (composite score, model-specific pain score) was based on the resource equation approach with focus in the 3 groups with osteotomy and different analgesia (min. number per group = 4; max. number per group = 8). For secondary endpoints such as water intake (ml) and BV/TV (bone volume fraction), power analysis was possible, calculated via G*Power 3.1 program and compared to the group sizes calculated via the resource equation approach. For more detailed information please see Animal Study Registry (Bf3R, Germany, 10.17590/asr.0000113).

The number of animals is either stated in the text, figure legend or indicated as a scatter dot plot in the graphs. Statistical analysis was carried out with GraphPad Prism V.7 software. As a first step, data sets were tested for Gaussian distribution using Shapiro-Wilk normality test (n < 8) or Kolmogorov-Smirnov normality test (n > 4). Data with n ≤ 4 were assumed as non-parametric. Only the body weight showed a Gaussian distribution compared to all other data sets. Therefore, the body weight is presented as mean ± SD and normalized to the initial body weight. To statistically compare the measured body weight to the initial body weight, one sample *t* test was performed (hypothetical mean = 100). One-way ANOVA with Bonferroni’s multiple comparisons test (selected pairs) was used to determine group differences. Changes over time were assessed with a repeated-measure two-way ANOVA with a Tukey’s multiple comparisons test. Normalization to baseline measurements was not useful in the other datasets. Where reasonable, baseline measurements are stated in the text or depicted in the graphs (time point = 0). Data with no Gaussian distribution are shown as median ± interquartile range. To determine statistical differences towards the baseline measurements, Wilcoxon signed rank test was used. As non-parametric statistical test for group differences, the Kruskal-Wallis test with Dunn’s multiple comparison test was applied for selected pairs. All important numbers and adjusted *p-*values are either stated in the text, the graphs or are listed in the Supplement.

## Supplementary information

Supplemental Information

## Data Availability

The authors declare that all data supporting the findings of this study are available within the paper and its supplementary information file. Further information on the study design and the protocols are available in the Animal Study Registry (Bf3R, Germany; 10.17590/asr.0000113). Further information are made available by the authors upon request.
