# Supplementary material for: Administration of Tramadol or Buprenorphine via the drinking water for post-operative analgesia in a mouse-osteotomy model
Source: Sci Rep. 2019 Jul 24;9:10749. doi: 10.1038/s41598-019-47186-5 (PMC6656891; doi:10.1038/s41598-019-47186-5)
Supplement: Supplementary file 1 — Supplemental Information [file 41598_2019_47186_MOESM1_ESM.pdf]

Supplemental Information

Administration of Tramadol or Buprenorphine via the drinking water for post-operatively analgesia in a mouse-osteotomy model

Paulin Jirkof[1#], Mattea Durst[1,2#], Robert Klopfleisch[3], Rupert Palme[4], Christa Thöne-Reineke[5], Frank Buttgereit[2,6], Katharina Schmidt-Bleek[7,8], Annemarie Lang[2,6,8]*

[1] Division of Surgical Research, University Hospital Zurich, University of Zurich, Zurich, Switzerland

[2] Charité – Universitätsmedizin Berlin, corporate member of Freie Universität Berlin, Humboldt-Universität zu Berlin, and Berlin Institute of Health, Department of Rheumatology and Clinical Immunology, Berlin, Germany

[3] Institute of Veterinary Pathology, Freie Universität Berlin, Germany

[4] Unit of Physiology, Pathophysiology and Experimental Endocrinology, Department of Biomedical Sciences, University of Veterinary Medicine, Vienna, Austria

[5] Institute of Animal Welfare, Animal Behavior and Laboratory Animal Science, Department of Veterinary Medicine, Freie Universität Berlin, Berlin, Germany

[6] German Rheumatism Research Centre (DRFZ) Berlin, a Leibniz Institute, Berlin, Germany

[7] Charité – Universitätsmedizin Berlin, corporate member of Freie Universität Berlin, Humboldt-Universität zu Berlin, and Berlin Institute of Health, Julius Wolff Institute and Center for Musculoskeletal Surgery, Berlin, Germany

[8] Charité – Universitätsmedizin Berlin, corporate member of Freie Universität Berlin, Humboldt-Universität zu Berlin, and Berlin Institute of Health Berlin Brandenburg Center for Regenerative Therapies, Berlin, Germany

[#] These authors contributed equally.

*Corresponding author: Annemarie Lang, Address: Charitéplatz 1, 10117 Berlin, Germany. Tel: (+49) 30 450 513450. Email: annemarie.lang@charite.de

Table S1: Food and water intake of the control groups (AN, DW)

	**Food intake (g)** Median (Min – Max)					**Water intake (ml)** Median (Min – Max)				
**Groups**	**0 h**	**24 h**	**48 h**	**72 h**	**96 h**	**0 h**	**24 h**	**48 h**	**72 h**	**96 h**
T_{low} **AN**	8.28 (7.63 – 8.93)	8.25 (2.4 – 14.1)	4.44 (3.9 – 5.0)	10.07 (5.4 – 14.7)	8.94 (4.5 – 13.4)	8.32 (8.1 – 8.6)	4.96 (1.3 – 8.6)	8.52 (7.1 – 10.0)	12.79 (11.3 – 14.3)	9.62 (9.6 – 9.6)
T_{high} **AN**	9.23 (8.7 – 9.8)	5.56 (5.0 – 6.0)	2.93 (0.3 – 5.5)	8.50 (7.2 – 9.8)	10.61 (10.3 – 10.9)	9.18 (8.3 – 10.1)	8.65 (6.0 – 11.3)	1.63 (0.9 – 2.4)	10.13 (9.4 – 10.8)	10.52 (9.9 – 11.2)
**Bup AN**	8.21 (7.3 – 9.1)	7.30 (7.0 – 7.6)	4.39 (2.4 – 6.4)	7.47 (6.4 – 8.5)	8.94 (7.3 – 10.6)	8.54 (8.2 – 8.9)	7.47 (6.4 – 8.6)	6.41 (6.0 – 6.8)	9.68 (9.5 – 9.8)	8.09 (8.1 – 8.1)
T_{low} **DW**	8.19 (8.0 – 8.4)	10.78 (8.6 – 13.0)	8.36 (7.8 – 8.9)	6.96 (6.5 – 7.4)	8.06 (5.2 – 10.9)	8.76 (8.3 – 9.2)	10.44 (8.4 – 12.5)	9.08 (5.8 – 12.4)	8.86 (8.8 – 8.9)	7.81 (7.8 – 7.8)
T_{high} **DW**	8.53 (8.2 – 8.9)	8.01 (7.6 – 8.4)	8.40 (6.5 – 10.4)	6.27 (2.7 – 9.8)	10.28 (7.2 – 13.3)	9.27 (8.2 – 10.3)	9.86 (4.7 – 15.1)	6.89 (5.3 – 8.5)	7.38 (6.9 – 7.8)	10.52 (10.8 – 10.3)
**Bup DW**	8.60 (7.3 – 9.9)	10.32 (9.9 – 10.7)	6.50 (6.3 – 6.7)	7.65 (7.6 – 7.8)	8.03 (7.2 – 8.8)	8.65 (8.3 – 9.0)	4.62 (2.3 – 7.0)	6.37 (4.5 – 8.3)	8.55 (7.3 – 9.8)	6.40 (6.1 – 6.7)

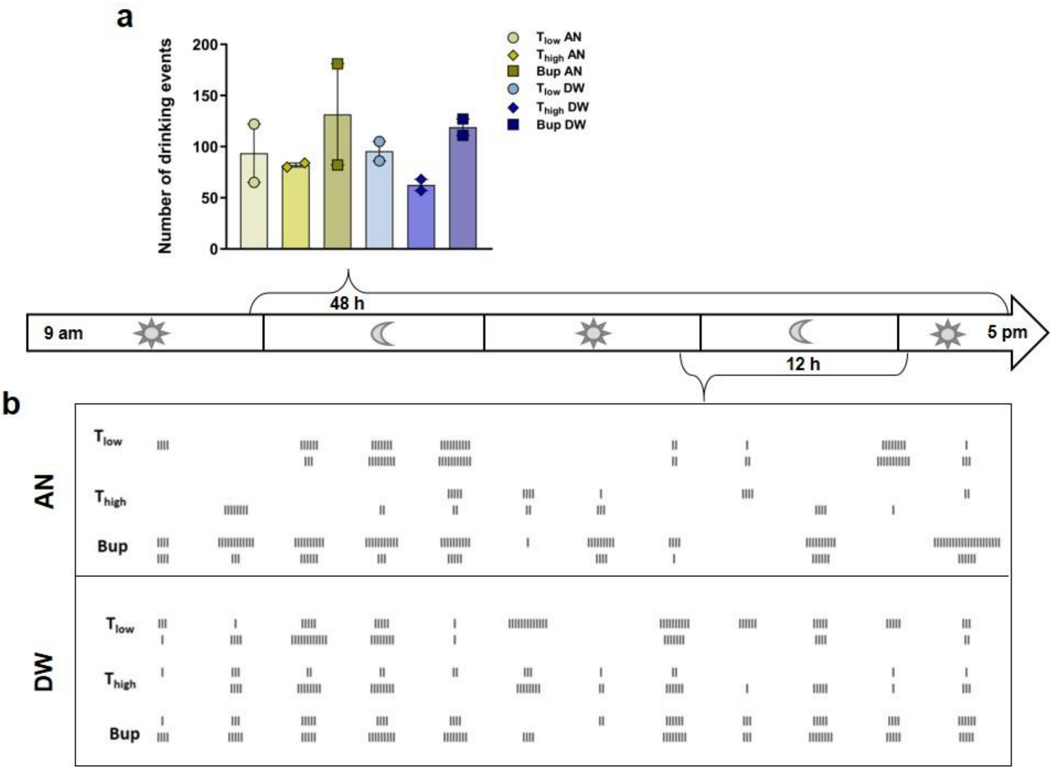

Figure S1: Drinking frequency of control (AN, DW) groups. (a) Total number of drinking events for 48 h was assessed via video recording. Data are shown for AN and DW groups as scatter dot plot and bar with median ± interquartile ranges (n = 2). (b) Depiction of the drinking events over 12 h between 36 h and 48 h post-osteotomy. Each line indicates one drinking event (n = 2).

Table S2: Exact *p*-values for differences between measured and initial body weight (one sample t test; hypothetical mean = 100)

	OT					AN					DW				
**Groups**	**24 h**	**48 h**	**72 h**	**96 h**	**120 h**	**24 h**	**48 h**	**72 h**	**96 h**	**120 h**	**24 h**	**48 h**	**72 h**	**96 h**	**120 h**
T_{low}	***< 0.001***	***< 0.001***	***< 0.001***	*0.70*	*0.120*	***0.020***	***0.020***	***0.031***	*0.270*	*0.870*	***0.001***	***0.031***	*0.970*	***0.020***	***0.040***
T_{high}	***< 0.001***	***< 0.001***	***< 0.001***	***< 0.001***	*0.310*	***0.01***	***0.005***	***<0.001***	***0.009***	*0.680*	***<0.001***	***0.002***	***0.005***	*0.230*	*0.560*
**Bup**	***0.005***	***<0.001***	***0.003***	*0.550*	*0.560*	***0.009***	***0.031***	*0.190*	*0.860*	*0.980*	*0.070*	*0.590*	*0.050*	*0.720*	***0.020***

Table S3: Adjusted *p*-values - Two-way ANOVA fold change body weight (Tukey's multiple comparisons test)

	Hours post-osteotomy	T_{low}	T_{high}	Bup
OT	24 vs. 48	0.8	>0.99	0.94
	24 vs. 72	**<0.001**	0.23	**0.02**
	24 vs. 96	**<0.001**	**<0.001**	**<0.001**
	24 vs. 120	**<0.001**	**<0.001**	**<0.001**
	48 vs. 72	**0.002**	0.13	**0.001**
	48 vs. 96	**<0.001**	**<0.001**	**<0.001**
	48 vs. 120	**<0.001**	**<0.001**	**<0.001**
	72 vs. 96	**<0.001**	**0.01**	0.05
	72 vs. 120	**<0.001**	**<0.001**	0.09
	96 vs. 120	0.46	**<0.001**	>0.99
AN	24 vs. 48	0.89	**0.01**	0.07
	24 vs. 72	**<0.001**	0.79	0.98
	24 vs. 96	**<0.001**	0.05	0.19
	24 vs. 120	**<0.001**	**<0.001**	0.35
	48 vs. 72	**0.003**	0.22	0.24
	48 vs. 96	**<0.001**	**<0.001**	**<0.001**
	48 vs. 120	**<0.001**	**<0.001**	**<0.001**
	72 vs. 96	**0.001**	**0.001**	0.05
	72 vs. 120	**0.009**	**<0.001**	0.12
	96 vs. 120	0.97	0.07	>0.99
DW	24 vs. 48	0.17	0.99	>0.99
	24 vs. 72	**<0.001**	0.26	0.84
	24 vs. 96	**<0.001**	**<0.001**	0.12
	24 vs. 120	**<0.001**	**<0.001**	0.17
	48 vs. 72	0.13	0.53	0.79
	48 vs. 96	**<0.001**	**<0.001**	0.1
	48 vs. 120	**<0.001**	**<0.001**	0.14
	72 vs. 96	0.11	0.07	0.66
	72 vs. 120	0.27	**<0.001**	0.75

Table S4: Exact *p*-values for differences between measured and initial facial expression and body composite score (Wilcoxon signed rank test; hypothetical median = 0)

	OT						AN					
**Groups**	**1 h**	**6 h**	**12 h**	**24 h**	**48 h**	**72 h**	**1 h**	**6 h**	**12 h**	**24 h**	**48 h**	**72 h**
T_{low}	***0.008***	***0.008***	***0.031***	***0.016***	*0.125*	*0.125*	*0.125*	*0.125*	*0.250*	*0.250*	*0.500*	*0.500*
T_{high}	***0.016***	***0.031***	*0.063*	*0.063*	*0.5*	*0.25*	*0.125*	*0.125*	*0.250*	*0.250*	*0.500*	-
**Bup**	*0.008*	*0.008*	*0.063*	***0.031***	*0.063*	*0.500*	*0.125*	*0.250*	-	*0.500*	*> 0.99*	-

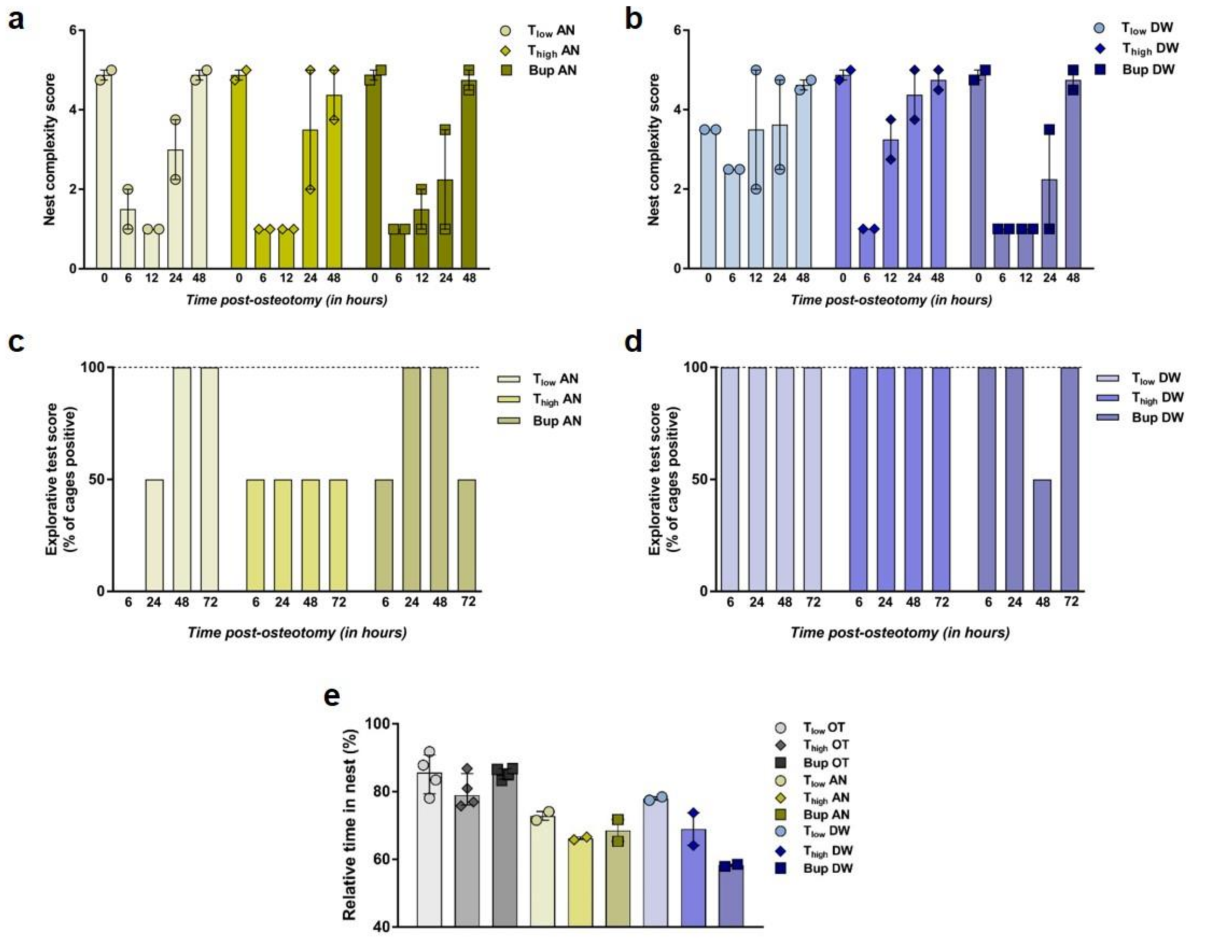

Figure S2: Nest complexity and explorative test scores of control (AN, DW) groups. (a, b) Nest complexity score are depicted for 0, 6, 12, 24 and 48 h post-osteotomy. Data are shown as scatter dot plot and bar with median ± interquartile range for n = 2 (cages). (c, d) The explorative test score is depicted for the OT groups as mean – percentage of cages that were determined positive for n = 2 (cages). (e) Relative time spent in nest for 48 h as assessed via video analysis. Data are shown as scatter dot plot and bar with median ± interquartile ranges (n = 2-4).

Table S5: Exact *p*-values for differences between measured and initial limp score (Wilcoxon signed rank test; hypothetical median = 0)

	OT				
**Groups**	**1 h**	**6 h**	**24 h**	**48 h**	**72 h**
T_{low}	***0.031***	*0.250*	*0.250*	*0.500*	*0.500*
T_{high}	***0.031***	*0.063*	*0.250*	*0.500*	*0.500*
**Bup**	*> 0.99*	*0.500*	*> 0.99*	*> 0.99*	*> 0.99*

Table S6: Overview on the different groups enrolled in the study

Group name and colour coding	Osteotomy groups (OT)			Control groups (AN, DW)					
	T_{low}	T_{high}	**Bup**	T_{low} AN	T_{low} DW	T_{high} AN	T_{high} DW	Bup AN	Bup DW
**Medication**	Tramadol		Buprenorphine	Tramadol				Buprenorphine	
**Dosage via drinking water 3d post-OP**	0.1 mg/ml	1 mg/ml	0.009 mg/ml	0.1 mg/ml		1 mg/ml		0.009 mg/ml	
**Mice number (n)**	8	8	8	4	4	4	4	4	4
**Medication pre-OP**	0.03 ml Clindamycin s.c. and Buprenorphine 0.1 ml s.c. (1h prä-OP)								
**Isoflurane anaesthesia**	yes			yes	no	yes	no	yes	no
**Euthansia after 2 weeks**	yes			no - reuse					

Table S7: Facial expression and body condition composite score adapted from Jirkof et al. [1]

Parameter	Specification	Scores
Facial expression		
**orbital tightening**	narrowing of the orbital area, a tightly closed eyelid, or an eye squeeze (orbital muscles around the eyes contracted)	**not present = 0**<br>**moderately = 1**<br>**severe = 2**
**ear position**	ears pulled back or rotate outwards and/or back, away from the face, space between the ears may appear wider	
Body condition		
**spontaneous behavior**	sudden movements, backwards movements, transient involuntary muscular contraction of any body part, kicking with hind paws, licking/biting the wound, highly aggressive, vocalization	
**posture**	hunched, arched back, crouched	
**coat condition**	ruffled, dirty, unkempt, piloerection, hair loss (alopecia)	
**eyes**	discharge	**not present = 0**<br>**present = 1**
**body condition**	sunken flanks, swollen areas, ascites	
**wound**	dirty, bloody, uncleaned, signs of self-injury, signs of inflammation or necrosis, i.e., unusual color (e.g., red, pale) or swollen	
**movement**	apathetic, sedated, decelerated, crawling, immobile, lameness, tiptoe gait	

Table S8: Limp score adapted from [2-4]

	Parameter	Specification	Scores
Limp Score	**Limping**	• normal use<br>• sporadic limping/hopping, complete ground contact<br>• limping, constant hopping<br>• partial non-use of limb<br>• complete lack of use	**0**<br>**1**<br>**2**<br>**3**<br>**4**
	**Dragging**	• normal use<br>• sporadic dragging of toes<br>• constant dragging of toes<br>• sporadic dragging of complete leg<br>• constant dragging of complete leg	**0**<br>**1**<br>**2**<br>**3**<br>**4**

References

1 Jirkof, P., Tourvieille, A., Cinelli, P. & Arras, M. Buprenorphine for pain relief in mice: repeated injections vs sustained-release depot formulation. *Laboratory animals*, doi:10.1177/0023677214562849 (2014).

2 Jimenez-Andrade, J. M. *et al.* Nerve growth factor sequestering therapy attenuates non-malignant skeletal pain following fracture. *Pain* **133**, 183-196, doi:10.1016/j.pain.2007.06.016 (2007).

3 Koewler, N. J. *et al.* Effects of a monoclonal antibody raised against nerve growth factor on skeletal pain and bone healing after fracture of the C57BL/6J mouse femur. *J Bone Miner Res* **22**, 1732-1742, doi:10.1359/jbmr.070711 (2007).

4 Minville, V., Laffosse, J. M., Fourcade, O., Girolami, J. P. & Tack, I. Mouse model of fracture pain. *Anesthesiology* **108**, 467-472 (2008).
